# Ferulic Acid Promotes Hematopoietic Stem Cell Maintenance in Homeostasis and Injury Through Diminishing Ferroptosis Susceptibility

**DOI:** 10.3390/antiox14091053

**Published:** 2025-08-27

**Authors:** Shuzhen Zhang, Yimin Zhang, Jiacheng Le, Kuan Yu, Xinliang Chen, Jun Chen, Mo Chen, Yiding Wu, Yang Xu, Song Wang, Chaonan Liu, Junping Wang, Changhong Du

**Affiliations:** 1 State Key Laboratory of Trauma and Chemical Poisoning, Institute of Combined Injury, Chongqing Engineering Research Center for Nanomedicine, College of Preventive Medicine, Army Medical University (Third Military Medical University), Chongqing 400038, China; zhangsz@tmmu.edu.cn (S.Z.); 13627664637@163.com (Y.Z.); yjc965381875o@163.com (J.L.); yukuan1205@163.com (K.Y.); 18323060623@163.com (X.C.); chenjun_amu@163.com (J.C.); chenmoi66@163.com (M.C.); wuyiding791@163.com (Y.W.); xyzq2023@163.com (Y.X.); gunnm1981@163.com (S.W.); naomiliu0306@163.com (C.L.); 2 Frontier Medical Training Brigade, Army Medical University (Third Military Medical University), Changji 831200, China

**Keywords:** ferulic acid, hematopoietic stem cell, ferroptosis, iron, ionizing radiation, myelosuppression

## Abstract

Redox balance is essential for maintenance of the hematopoietic stem cell (HSC) pool, which ensures the lifelong hematopoiesis. However, oxidative attack induced by various physiopathological stresses always compromises HSC maintenance, while there remains lack of safe and effective antioxidative measures combating these conditions. Here, we show that ferulic acid (FA), a natural antioxidant abundantly present in Angelica sinensis which is a traditional Chinese herb commonly used for promotion of blood production, distinctively and directly promotes HSC maintenance and thereby boosts hematopoiesis at homeostasis, whether supplemented over the long term in vivo or in HSC culture ex vivo. Using a mouse model of acute myelosuppressive injury induced by ionizing radiation, we further reveal that FA supplementation effectively safeguards HSC maintenance and accelerates hematopoietic regeneration after acute myelosuppressive injury. Mechanistically, FA diminishes ferroptosis susceptibility of HSCs through limiting the labile iron pool (LIP), thus favoring HSC maintenance. In addition, the LIP limitation and anti-ferroptosis activity of FA is independent of nuclear-factor erythroid 2-related factor 2 (NRF2), probably relying on its iron-chelating ability. These findings not only uncover a novel pharmacological action and mechanism of FA in promoting HSC maintenance, but also provides a therapeutic rationale for using FA or FA-rich herbs to treat iron overload- and ferroptosis-associated pathologies such as acute myelosuppressive injury.

## 1. Introduction

Hematopoiesis, also termed as blood production, is a vital and constant function through which all blood and immune cells are produced throughout life to support oxygen transport, immunological responses and hemostasis. Generally, adult hematopoiesis follows a hierarchical developmental process in the bone marrow (BM) whereby hematopoietic stem cells (HSCs) differentiate into mature blood cells through a series of increasingly lineage-restricted progenitors [1,2]. To ensure lifelong hematopoiesis, HSCs are tightly maintained by a range of intrinsic and extrinsic mechanisms, among which the antioxidative defense is particularly crucial due to its essentiality in the maintenance of genomic integrity, quiescence and functionality of HSCs [3]. However, the sporadic oxidative attack over life, or the devastating oxidative attack imposed by myelosuppressive insults such as irradiation, chemotherapeutics and infection, will profoundly compromise HSC maintenance, leading to an increased risk of developing BM aplasia with age or life-threatening myelosuppression, respectively [3,4,5]. Despite substantial efforts devoted to investigating the underlying mechanisms and searching for therapeutic strategies, there remains a lack of safe and effective antioxidative measures combating these conditions.

Dozens of traditional Chinese medicine formulas have been used for promotion of hematopoiesis for centuries, with Angelica sinensis being used as a common herb in these formulas [6]. Angelica sinensis has also been widely used as a health food or dietary supplement for women’s care [7]. Unfortunately, the cellular and molecular bases of the hematopoiesis-promoting effect of Angelica sinensis remain largely unknown. Recently, ferulic acid (FA), a major chemical component identified in Angelica sinensis, has attracted significant research interest. As a family of phenolic acids, FA exhibits a variety of bioactive properties, particularly notable for its antioxidative effects [8]. Although the action of FA in hematopoiesis remains undefined, FA supplementation has been documented to alleviate irradiation-induced long-term hematopoietic injuries at least partially through attenuating oxidative stress [9,10,11]. In light of these properties, it is plausible that FA may favor HSC maintenance during homeostasis through preventing oxidative attack. Also, it seems reasonable that FA would confer HSC protection against acute myelosuppressive injury, which is frequently encountered in clinics by cancer patients receiving radiotherapy and chemotherapy [12], through mitigating oxidative attack. And if so, FA may serve as an important active compound contributing to the hematopoiesis-promoting effect of Angelica sinensis and as a natural product may represent a promising therapeutic for preventing and treating oxidative stress-associated hematopoietic disorders, such as BM failure [5], HSC aging [13], and clonal hematopoiesis [14].

In this study, we determined the action of FA in hematopoiesis as well as the underlying cellular and molecular mechanisms. We revealed that FA distinctively and directly promotes HSC maintenance and thereby boosts hematopoiesis in both homeostasis and injury. Mechanistically, FA limits labile iron pool (LIP) to diminish ferroptosis susceptibility of HSCs, thus favoring HSC maintenance. These findings unmask a previously unknown action of FA in promoting HSC maintenance and suggest FA as a promising therapeutic candidate for acute myelosuppressive injury.

## 2. Materials and Methods

### 2.1. Animals

Wild-type C57BL/6 (CD45.2^+^) mice and C57BL/6Smoc-Ptprc^em1(K302E)Smoc^ (CD45.1^+^) mice were, respectively, purchased from Beijing HFK Bioscience Co., Ltd. (Beijing, China) and Shanghai Model Organisms Center, Inc. (Shanghai, China). All mice used were male, age-matched (8–10 weeks), and background-matched. Mice were kept in a pathogen-free facility at the Army Medical University under consistent environmental conditions, including a humidity level of (50  ±  5)%, a temperature of (24  ±  1) °C, a 12 h light/dark cycle, and free access to food and water. Animal experiments performed were approved by the Animal Care Committee of the Army Medical University and conducted according to the institutional guidelines.

### 2.2. Irradiation

Mice were exposed to a single dose of 5 or 7.5 Gy total body irradiation as indicated in a unilateral radiation field using a ^60^Co γ-ray source (Irradiation Center, Army Medical University, Chongqing, China), as we previously described [15]. The dose rate ranged from 92.8 to 95.5 cGy/min.

### 2.3. Pharmacological Treatment

For short-term FA supplementation, C57BL/6 mice received daily intraperitoneal injections of FA (50 mg/kg body weight; MedChem Express, Monmouth Junction, NJ, USA) for 7 consecutive days. For long-term FA supplementation, C57BL/6 mice received daily intraperitoneal injections of FA (50 mg/kg body weight) for 30 consecutive days. For imidazole ketone erastin (IKE; MedChem Express) treatment, mice received daily intraperitoneal injections of IKE at a dose of 40 mg/kg for 2 weeks from the second week of long-term FA supplementation. To test the protective effects of FA against acute myelosuppressive injury, C57BL/6 mice received daily intraperitoneal FA injections (50 mg/kg body weight) for 3 days prior to irradiation and continuing for 7 days after irradiation.

### 2.4. Hematological Parameter Test

Hematological parameters of mice were determined as we previously reported [16]. Briefly, 20 μL peripheral blood (PB) were collected from mouse tail veins and immediately diluted in 1% EDTA solution. Complete blood counts, including white blood cells (WBCs), red blood cells (RBCs), and platelets, were quantified using an automated Sysmex XT-2000i hematology analyzer (Sysmex Corporation, Kobe, Japan).

### 2.5. HSC Culture

HSCs were cultured using an established protocol that maintains functional properties ex vivo [17]. Briefly, sorted HSCs were plated in fibronectin-coated 24-well plates in a defined culture medium composed of F12 medium, murine recombinant thrombopoietin (100 ng/mL), murine recombinant stem cell factor (10 ng/mL; both PeproTech, Rocky Hill, NJ, USA), 1% penicillin/streptomycin/glutamine, 1% transferrin, 10 mM HEPES (all Thermo Fisher Scientific, Carlsbad, CA, USA), and 1 mg/mL polyvinyl alcohol (Sigma-Aldrich, St. Louis, MO, USA). Cultures were maintained at 37 °C in 5% CO_2_. Viable cell numbers were quantified every 48 h using a Countess™ II FL Automated Cell Counter (Thermo Fisher Scientific), with dead cells being excluded by trypan blue (Thermo Fisher Scientific). For FA or FeSO_4_ treatment, HSCs were cultured in the presence of 2–5 μM FA or 50–200 μM FeSO_4_ (both MedChem Express) as indicated. The HSC pool maintenance was assessed following 10 days of culture.

### 2.6. Flow Cytometry

#### 2.6.1. Cell Preparation and Sorting

BM cells were harvested from femurs and tibias by flushing with PBS, followed by erythrocyte depletion using a red cell lysis buffer (StemCell Technologies, Vancouver, BC, Canada). For phenotypic analysis of hematopoietic cells, a lineage cocktail including CD3ε, Ter-119, Mac-1, Gr-1 and B220 (all eBioscience, San Diego, CA, USA) was used. HSCs, myeloid progenitors, common myeloid progenitors (CMPs), granulocyte/monocyte progenitors (GMPs), megakaryocyte–erythrocyte progenitors (MEPs), common lymphoid progenitors (CLPs), myeloid cells, B cells, and T cells were analyzed using monoclonal antibodies as indicated. The detailed information of phenotypic markers for each hematopoietic cell population can be found in Table S1. Cells were stained with antibodies in Flow Cytometry Staining Buffer (eBioscience) for 30 min at 4 °C, washed, and analyzed on a Sony ID7000™ Spectral Cell Analyzer. HSCs were isolated using a Sony MA900 cell sorter (both Sony Biotechnology, Tokyo, Japan). Data were processed with FlowJo V10.8.1 software (Treestar Inc., San Carlos, CA, USA).

#### 2.6.2. Cytoplasmic Protein Detection

For ferritin detection, BM cells were first surface-stained with HSC markers in Flow Cytometry Staining Buffer as described above, then fixed using IC Fixation Buffer (eBioscience) for 20 min at room temperature. Cells were permeabilized with Permeabilization Buffer (eBioscience) containing an anti-ferritin antibody (Abcam, Cambridge, UK) for 45 min at room temperature. After washing, cells were stained with a fluorescent dye-conjugated secondary antibody (Thermo Fisher Scientific) for another 30 min at room temperature prior to flow cytometric analysis.

#### 2.6.3. Nuclear Protein Detection

For nuclear-factor erythroid 2-related factor 2 (NRF2) detection, BM cells were first surface-stained with HSC markers in Flow Cytometry Staining Buffer and washed. Cells were then fixed with 1 mL Foxp3 Fixation/Permeabilization working solution (eBioscience) for 30 min at room temperature, followed by intracellular staining with anti-NRF2 antibody (Thermo Fisher Scientific) in permeabilization buffer for 30 min at room temperature. After washing, cells were stained with a fluorescent dye-conjugated secondary antibody (Thermo Fisher Scientific) for another 30 min at room temperature prior to flow cytometric analysis. Antibody details are provided in Table S2.

### 2.7. Transplantation Assay

Competitive transplantation assay was performed using 1 × 10^6^ BM cells (CD45.2) from either control or FA-treated mice mixed with an equal number of competitor BM cells (CD45.1). The cell mixture was transplanted via tail vein injection into lethally irradiated (10 Gy split dose) CD45.1 recipient mice as previously described [16]. PB was collected from tail veins at 16 weeks post-transplantation for analysis of multi-lineage reconstitution (granulocyte/monocyte, B-cell, and T-cell populations) using a Sony ID7000™ Spectral Cell Analyzer. Donor chimerism was evaluated using the antibody panel detailed in Table S2.

### 2.8. Colony Assay

Colony assay was performed as we previously reported [18]. Briefly, 1000 hematopoietic stem and progenitor cell (HSPC)-enriched Lineage^−^ cells were sorted from HSC culture supplemented with or without FA and plated into methylcellulose medium (STEMCELL Technologies) according to the manufacturer’s instructions. Colonies were assessed 12 days later using an inverted light microscopy.

### 2.9. RNA-Sequencing (RNA-Seq)

HSCs were flow-sorted from either vehicle or l-FA treated mice, followed by RNA extraction using a RNeasy Micro Kit (QIAGEN, Hilden, Germany) according to the manufacturer’s protocol. concentration and purity were determined by NanoDrop ND-1000 (Thermo Fisher Scientific). The RNA fragments were copied into first strand cDNA using reverse transcriptase and several templateless C nucleotides. The oligonucleotide primers were then hybridized with poly(C) to form second strand cDNA. cDNA libraries were prepared using the KAPA Hyper Prep Kit (Kapa Biosystems, Wilmington, MA, USA) according to the manufacturer’s protocol. The resulting libraries were then purified and quantified and validated by Qubit^®^ 3.0 Fluorometer (Thermo Fisher Scientific) and Agilent 2100 Bioanalyzer (Agilent, Santa Clara, CA, USA), followed by sequencing on the Illumina NovaSeq 6000 platform (Illumina, San Diego, CA, USA). Differently expressed genes were screened by DEseq2 using a fold change > 2.0 and adjusted *p* value < 0.05. Kyoto Encyclopedia of Genes and Genomes (KEGG) pathway enrichment analysis were then performed based on the differentially expressed genes. Gene set enrichment analysis (GSEA V4.0.3) was performed using GSEA software based on Molecular Signatures Database v6.0 (http://software.broadinstitute.org/gsea/msigdb, accessed on 20 May 2025) [19].

### 2.10. Ferroptosis Assays

Ferroptosis assays were performed as we previously reported [16]. Briefly, BMCs were first surface-stained with HSC markers in Flow Cytometry Staining Buffer as described above, followed by thorough washing. For cell death analysis, cells were incubated with a 7-amino-actinomycin D (7-AAD) staining solution (Biolegend, San Diego, CA, USA) at room temperature for 15 min. For lipid peroxidation analysis, cells were stained in pre-warmed (37 °C) PBS in the presence of 10 mM LiperFluo (Dojindo Molecular Technologies, Kumamoto, Japan) at 37 °C for 30 min. After washing, cells were immediately analyzed by flow cytometry.

### 2.11. Ferroptosis Susceptibility Assay

Ferroptosis susceptibility assay was performed as we previously reported [16]. To analyze the ferroptosis susceptibility of HSCs in vivo, 5000 freshly sorted HSCs were sorted into 24-well plates and treated with 5 μM erastin (MedChem Express) in complete HSC medium. After incubation for 48 h, HSC ferroptosis was quantified by parallel staining with LiperFluo and 7-AAD as described above.

### 2.12. LIP Measurement

LIP in HSCs was measured as we previously reported [18]. Briefly, BM cells were first surface-stained with HSC markers in Flow Cytometry Staining Buffer as described above, followed by thorough washing. LIP was measured by incubating cells in pre-warmed (37 °C) PBS in the presence of 2 μM FerroOrange (Dojindo Molecular Technologies) for 30 min at 37 °C. After washing, cells were analyzed by flow cytometry.

### 2.13. Immunofluorescence

Freshly isolated HSCs were stained with 5 μM BODIPY 581/591 C11 (Thermo Fisher Scientific) or 2 μM FerroOrange for 30 min at 37 °C. Cells were then washed with pre-warmed (37 °C) HBSS, resuspended in 10 μL and immediately transferred to poly-L-lysine-coated slides. Then, cells were photographed using a Zeiss LSM980 NLO confocal microscope (Carl Zeiss, Jena, Germany) as soon as possible.

### 2.14. Hematoxylin and Eosin Staining

Femurs were fixed in 4% formaldehyde in PBS for 24 h at 4 °C, followed by decalcification in 10% EDTA-PBS buffer. After being embedded in paraffin, 5 μm-thick longitudinal sections were cut using a Leica RM2245 microtome (Leica, Wetzlar, Germany) and mounted on poly-L-lysine-coated slides. Sections were stained with hematoxylin and eosin following standard protocols. Histological evaluation was performed using a PreciPoint M8 digital microscope (PreciPoint, Freising, Germany) equipped with a 20× objective.

### 2.15. Statistical Analysis

Statistical analysis was performed using GraphPad Prism 9.0 software (GraphPad Software, La Jolla, CA, USA). Each experiment was repeated independently at least three times. Sample sizes and numbers of independent experiments for each figure are denoted as *n* in the figure legends. All data are represented as mean ± standard deviation (SD). Student’s *t*-test was used to compare differences between two groups, while one-way analysis of variance (ANOVA) was performed to compare differences among multiple groups followed by Tukey’s *post hoc* comparison test. Survival data from each group were analyzed using the Kaplan–Meier method, and the significance of the differences were tested via the log-rank test (Mantel–Cox). *p* < 0.05 was considered statistically significant.

## 3. Results

### 3.1. Short-Term FA Supplementation Has No Impact on Homeostatic Hematopoiesis

**Figure 1:**
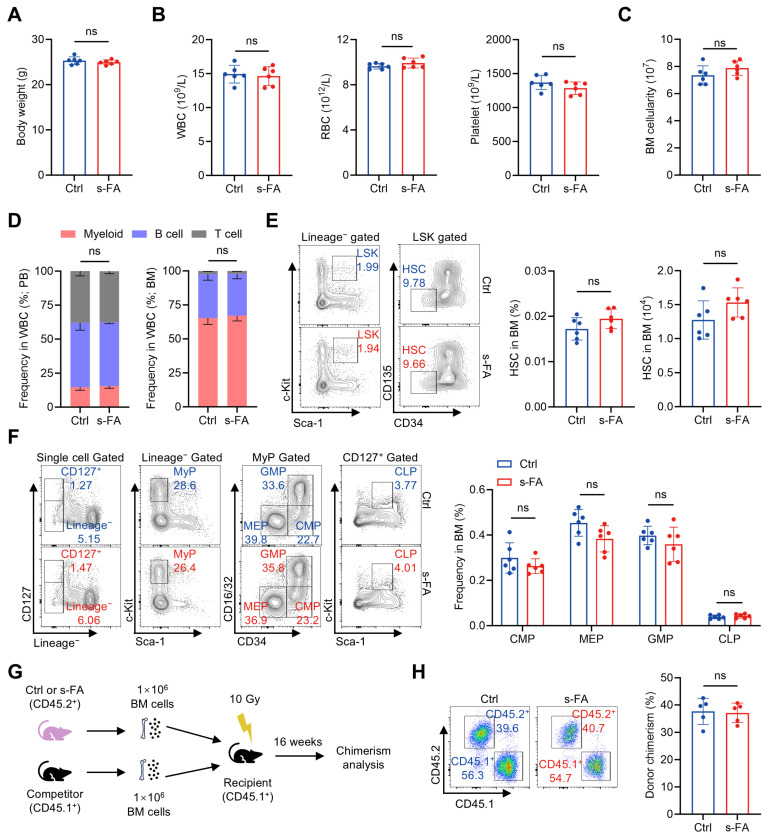
Short-term FA administration has no impact on homeostatic hematopoiesis. (**A**) Body weights of control (Ctrl) and s-FA-treated mice (*n* = 6). (**B**) WBC, RBC and platelet counts in PB of Ctrl and s-FA-treated mice (*n* = 6). (**C**) BM cellularity of Ctrl and s-FA-treated mice (*n* = 6). (**D**) Proportions of myeloid, B and T cells in PB and BM of Ctrl and s-FA-treated mice (*n* = 6). (**E**) Flow cytometric quantification of HSC frequency and number in the BM of Ctrl and s-FA-treated mice (*n* = 6). (**F**) Flow cytometric quantification of CMP, GMP, MEP and CLP frequencies in the BM of Ctrl and s-FA-treated mice (*n* = 6). (**G**) Scheme for competitive BM transplantation. (**H**) Flow cytometric quantification of donor chimerism in PB of recipient mice (*n* = 5). Data represent mean ± SD. ns, no significance. Two-tailed unpaired Student’s *t*-test.

To evaluate the influence of FA on hematopoietic system, we initially administered mice with a short-term FA (s-FA) supplementation regimen (50 mg/kg body weight, once daily for 7 consecutive days). s-FA supplementation was tolerated by mice, as no apparent influence on body weight was observed (Figure 1A). With respect to the hematopoietic system, we found that the counts of WBCs, RBCs and platelets in PB (Figure 1B), the cellularity of BM (Figure 1C), as well as the proportions of myeloid, B and T cells in the WBC compartment (Figure 1D), were all not affected by s-FA supplementation. At the HSPC level, the frequency and number of HSCs (Figure 1E), as well as the frequencies of hematopoietic progenitor cells including CMPs, GMPs, MEPs and CLPs (Figure 1F), in the BM of s-FA-treated mice were also comparable with those of control mice. To further determine the maintenance of functional HSCs, competitive BM transplantation was performed as illustrated in Figure 1G. It was detected that the donor chimerism of BM cells from s-FA-treated mice was comparable with that from control mice 16 weeks after transplantation (Figure 1H), when the vast majority of blood cells are produced by donor HSCs [16], reflecting unaffected maintenance of functional HSCs following s-FA supplementation. These data show that s-FA supplementation has no gross impact on homeostatic hematopoiesis.

### 3.2. Long-Term FA Supplementation Distinctly Promotes HSC Maintenance

**Figure 2:**
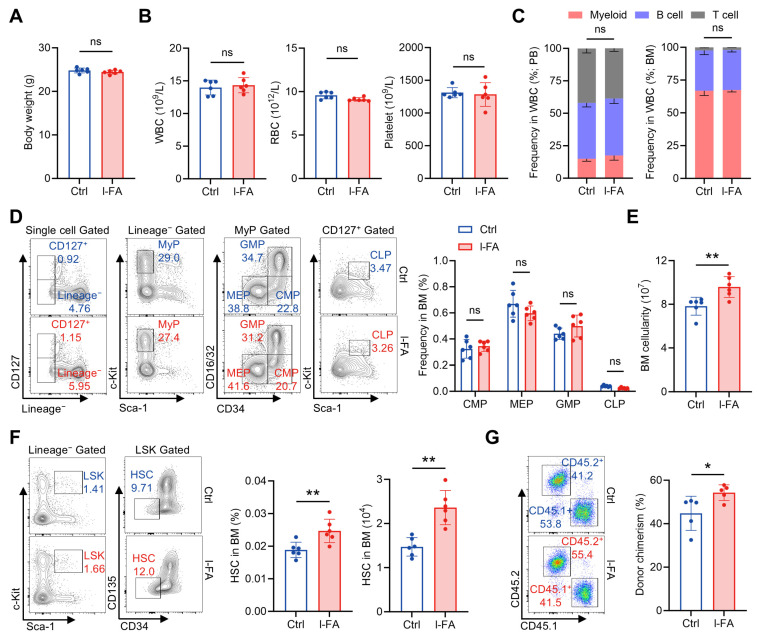
Long-term FA administration distinctively favors HSC maintenance. (**A**) Body weights of Ctrl and l-FA-treated mice (*n* = 6). (**B**) WBC, RBC and platelet counts in PB of Ctrl and l-FA-treated mice (*n* = 6). (**C**) Proportions of myeloid, T and B cells in PB and BM of Ctrl and l-FA-treated mice (*n* = 6). (**D**) Flow cytometric quantification of CMP, GMP, MEP and CLP frequencies in the BM of Ctrl and l-FA-treated mice (*n* = 6). (**E**) BM cellularity of Ctrl and l-FA-treated mice (*n* = 6). (**F**) Flow cytometric quantification of HSC frequency and number in the BM of Ctrl and l-FA-treated mice (*n* = 6). (**G**) Flow cytometric quantification of donor chimerism in PB of recipient mice (*n* = 5). Data represent mean ± SD. * *p* < 0.05, ** *p* < 0.01, ns, no significance. Two-tailed unpaired Student’s *t*-test.

Then, we interrogated whether a long-term FA (l-FA) supplementation regimen (50 mg/kg body weight, once daily for 30 consecutive days) would affect the hematopoietic system. l-FA supplementation was tolerated by mice as well, as reflected by no apparent influence on the body weight (Figure 2A). Comprehensive analysis of the hematopoietic system demonstrated no significant differences between l-FA-treated mice and control mice across the counts of WBCs, RBCs and platelets in PB (Figure 2B), the proportions of myeloid, B and T cells in the WBC compartment (Figure 2C), as well as the frequencies of CMPs, GMPs, MEPs and CLPs in the BM (Figure 2D). However, the BM cellularity was significantly increased in l-FA-treated versus control mice (Figure 2E), indicating enhanced BM hematopoiesis. Moreover, we also noticed that the frequency and number of HSCs were remarkably increased after l-FA supplementation (Figure 2F). Competitive BM transplantation further showed superior donor chimerism by l-FA-treated HSCs than that by control HSCs at 16 weeks after transplantation (Figure 1G), verifying enhanced maintenance of functional HSCs following l-FA supplementation. These data indicate that l-FA supplementation distinctively promotes HSC maintenance and thereby boosts BM hematopoiesis.

### 3.3. FA Directly Promotes HSC Maintenance Ex Vivo

**Figure 3:**
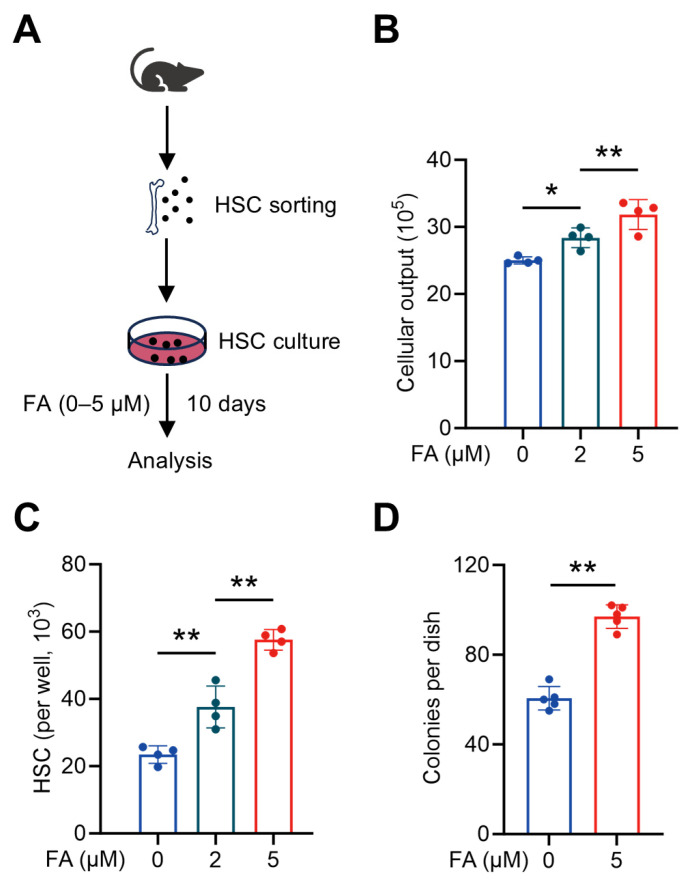
FA directly promotes HSC maintenance ex vivo. (**A**) Scheme for ex vivo HSC culture. (**B**) Cellular outputs of HSCs cultured with different concentrations of FA (*n* = 4). (**C**) HSC numbers in the cultures supplemented with different concentrations of FA (*n* = 4). (**D**) Colony numbers of per 103 lineage– cells sorted from HSC cultures supplemented with or without FA (*n* = 5). Data represent mean ± SD. * *p* < 0.05, ** *p* < 0.01. One-way ANOVA unless stated otherwise. Two-tailed unpaired Student’s *t*-test (**D**).

Next, we tested whether FA could directly promote HSC maintenance. To this end, we employed an established ex vivo culture system capable of maintaining HSC self-renewal and promoting functional HSC expansion [17], and supplemented the HSC culture with different concentrations of FA (Figure 3A). It was found that FA significantly boosted the cellular output of HSC culture within the concentration range of 2 to 5 μM in a dose-dependent manner (Figure 3B). Meanwhile, as did the results in vivo, FA also significantly and dose-dependently promoted the expansion of HSCs ex vivo (Figure 3C), reinforcing that the enhanced HSC maintenance underlies the boosted hematopoiesis. To further determine the maintenance of functional HSCs ex vivo, we performed a colony assay by plating HSPC-enriched Lineage^−^ cells sorted from HSC culture into a methylcellulose medium. It was observed that more colonies were formed by Lineage^−^ cells sorted from the HSC culture supplemented with FA (Figure 3D), confirming that functional HSCs were expanded in the presence of FA ex vivo. Thus, FA is capable of directly promoting HSC maintenance, thereby boosting hematopoiesis.

### 3.4. FA Diminishes Ferroptosis Susceptibility of HSCs

**Figure 4:**
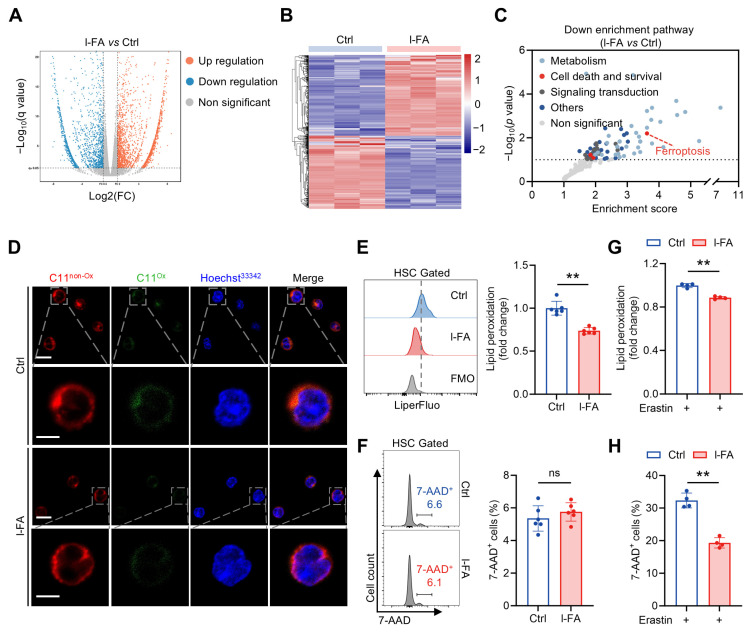
FA diminishes ferroptosis susceptibility of HSCs. (**A**,**B**) Transcriptomic profiling of BM HSCs of Ctrl and l-FA-treated mice. (**C**) Downregulated signaling pathways as revealed by KEGG pathway enrichment analysis in BM HSCs of l-FA-treated versus Ctrl mice. (**D**) Membrane lipid peroxidation as reflected by BODIPY 581/591 C11 staining in BM HSCs of Ctrl and l-FA-treated mice. Scale bar, 10 μm (upper panel). Scale bar, 5 μm (down panel). (**E**,**F**) Flow cytometric quantification of membrane lipid peroxidation (**E**) and cell death (**F**) in BM HSCs of Ctrl and l-FA-treated mice (*n* = 6). (**G**,**H**) Ferroptosis susceptibility analysis of BM HSCs in Ctrl and l-FA-treated mice (*n* = 4). Data represent mean ± SD. ** *p* < 0.01, ns, no significance. Two-tailed unpaired Student’s *t*-test.

To elucidate the mechanism by which FA directly promotes HSC maintenance, RNA-seq analysis of HSCs from mice with l-FA supplementation was performed, and we observed profound transcriptional changes compared to control mice (Figure 4A,B). Interestingly, among the top altered signaling pathways, we noticed a predominant downregulation of the ferroptosis signaling pathway in HSCs of l-FA-treated mice compared to control mice (Figure 4C). As known, ferroptosis is an oxidative form of cell death characterized by overwhelming membrane lipid peroxidation and rupture of the plasma membrane [20]. Meanwhile, HSCs are recently demonstrated to be ferroptosis vulnerable [21], and ferroptosis will impair HSC maintenance in both homeostasis and injury [16,18]. In correspondence, BODIPY 581/591 C11 staining (Figure 4D) and LiperFluo staining (Figure 4E) showed a remarkable reduction in membrane lipid peroxidation in HSCs of l-FA-treated mice compared to control mice. However, 7-AAD staining reflected negligible and comparable HSC death between these two groups (Figure 4F). These lines of evidence inform that FA diminishes ferroptosis susceptibility of HSCs. In keeping with this, l-FA-treated HSCs were more resistant to ferroptosis induced by a canonical ferroptosis inducer erastin (Figure 4G,H). Similarly to the results in vivo, although FA supplementation had no apparent influence on HSC death ex vivo (Figure S1A), it remarkably mitigated lipid peroxidation in HSCs (Figure S1B). Moreover, FA supplementation also significantly attenuated erastin-induced HSC ferroptosis ex vivo (Figure S1C,D).

**Figure 5:**
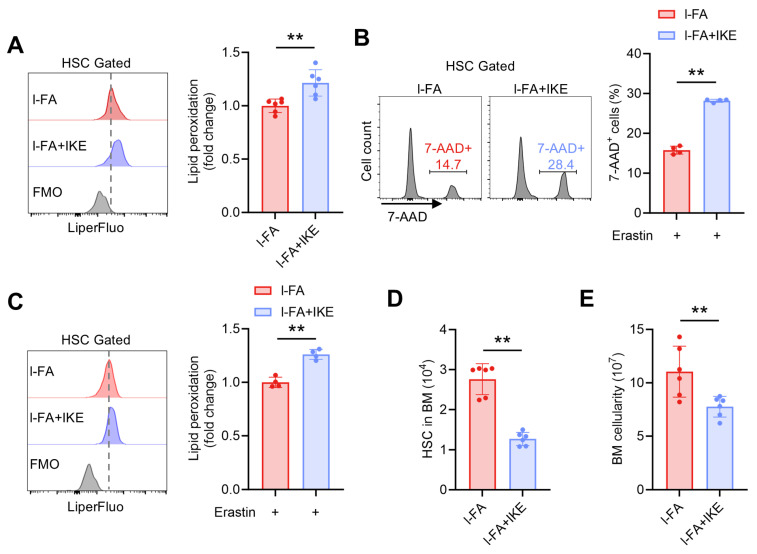
IKE administration restores ferroptosis susceptibility and maintenance of HSCs in l-FA-treated mice. (**A**) Flow cytometric quantification of membrane lipid peroxidation in BM HSCs of l-FA-treated mice with or without IKE administration (*n* = 6). (**B**,**C**) Ferroptosis susceptibility analysis of BM HSCs in l-FA-treated mice with or without IKE administration (*n* = 4). (**D**) HSC numbers in the BM of l-FA-treated mice with or without IKE administration (*n* = 6). (**E**) BM cellularity of l-FA-treated mice with or without IKE administration (*n* = 6). Data represent mean ± SD. ** *p* < 0.01. Two-tailed unpaired Student’s *t*-test.

To further validate the involvement of diminished ferroptosis susceptibility in the promotion of HSC maintenance by FA, l-FA-treated mice were administered with IKE, a potent ferroptosis inducer with demonstrated in vivo efficacy [22]. It was observed that IKE administration markedly restored the ferroptosis susceptibility of HSCs in l-FA-treated mice, as demonstrated by increased lipid peroxidation (Figure 5A) and susceptibility to erastin-induced ferroptosis of HSCs (Figure 5B,C). Finally, the enhanced HSC maintenance (Figure 5D) and increased BM cellularity (Figure 5E) in l-FA-treated mice were both abrogated by IKE administration. Taken together, these data demonstrate that FA directly promotes HSC maintenance through diminishing ferroptosis susceptibility of HSCs.

### 3.5. FA Limits Labile Iron (Fe^2+^) Pool in HSCs Independently of NRF2

**Figure 6:**
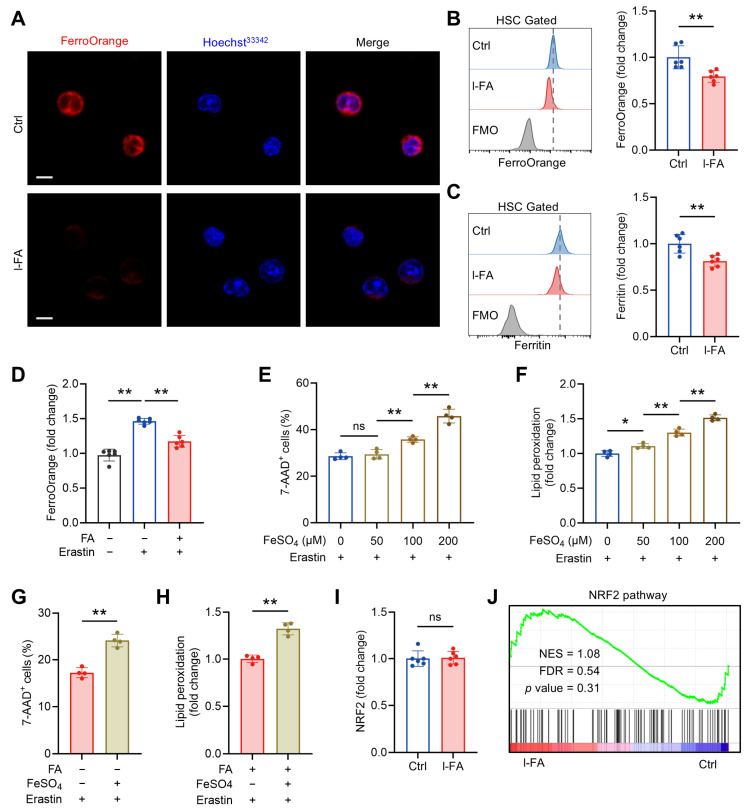
FA limits labile iron (Fe^2+^) pool in HSCs independently of NRF2. (**A**) Analysis of LIP size by FerroOrange staining in BM HSCs of Ctrl and l-FA-treated mice. Scale bar, 10 μm. (**B**) Flow cytometric quantification of LIP size in BM HSCs of Ctrl and l-FA-treated mice (*n* = 6). (**C**) Flow cytometric quantification of ferritin expression in BM HSCs of Ctrl and l-FA-treated mice (*n* = 6). (**D**) LIP size in HSCs cultured with FA and/or erastin (*n* = 6). (**E**,**F**) Ferroptosis analysis of HSCs cultured with different concentrations of FeSO_4_ (*n* = 4). (**G**,**H**) Ferroptosis analysis of HSCs cultured with FA and/or FeSO_4_ (*n* = 4). (**I**) Flow cytometric quantification of NRF2 expression in BM HSCs of Ctrl and l-FA-treated mice (*n* = 6). (**J**) GSEA of NRF2 pathway in BM HSCs of l-FA-treated versus Ctrl mice. Data represent mean ± SD. * *p* < 0.05, ** *p* < 0.01, ns, no significance. Two-tailed unpaired Student’s *t*-test unless stated otherwise. One-way ANOVA (**D**–**F**). NES, normalized enrichment score; FDR, false discovery rate.

Ferroptosis is an iron-dependent form of cell death, tightly associated with intracellular LIP size [20]. Intriguingly, FA is demonstrated to have the capacity to chelate Fe^2+^ [23]. In light of the above, we asked whether the anti-ferroptosis activity of FA is related to LIP limitation. To this end, we stained HSCs with a Fe^2+^ chemisensor, FerroOrange, to quantify intracellular LIP size. In line with a recent study [24], HSCs harbor well-detectable LIP, whereas the LIP size significantly declined in HSCs of l-FA-treated versus control mice (Figure 6A,B). Correspondingly, HSCs from l-FA-treated mice exhibited an iron-limited status characterized by reduced protein abundance of ferritin (Figure 6C), which is a major iron storage protein positively correlating with LIP size [25]. Ex vivo, FA supplementation also significantly reduced the LIP size in cultured HSCs (Figure S2A), accompanied by decreased protein abundance of ferritin (Figure S2B). In addition, treatment of cultured HSCs with erastin resulted in remarkably increased LIP size in HSCs, which was significantly attenuated by FA supplementation (Figure 6D). Further, we observed that FeSO_4_ synergistically enhanced erastin-mediated HSC ferroptosis in a concentration-dependent manner, with a significant difference at the concentration above 50 μM (Figure 6E,F). However, supplementation with 50 μM FeSO_4_, which has nearly no influence on erastin-induced HSC ferroptosis, abrogated the protective effect of FA against erastin-induced HSC ferroptosis (Figure 6G,H). Thus, FA is capable of limiting LIP in HSCs, thereby diminishing ferroptosis susceptibility of HSCs.

NRF2 is a transcription factor that responds to oxidative stress by modulating several pathways involved in ferroptosis, including those related to glutathione production, antioxidative responses, and iron homeostasis [26]. Given the essential role of NRF2 in HSC maintenance [27] and that FA can activate NRF2 signaling to counteract oxidative stress in different experimental models [28,29,30,31], we further tested whether NRF2 is implicated in the LIP limitation and anti-ferroptosis activity of FA in HSCs. Both in vivo and ex vivo, FA treatment had no obvious impact on nuclear NRF2 protein expression (Figure 6I and Figure S2C) and NRF2 signaling as reflected by GSEA (Figure 6J). Thus, NRF2 is dispensable for the LIP limitation and anti-ferroptosis activity of FA in HSCs. Taken together, these results consolidate that FA diminishes ferroptosis susceptibility of HSCs through limiting LIP size. In addition, the LIP limitation activity of FA in HSCs is independent of NRF2 activation, probably relying on its iron-chelating ability.

### 3.6. FA Safeguards HSC Maintenance and Accelerates Hematopoietic Regeneration After Acute Myelosuppressive Injury

**Figure 7:**
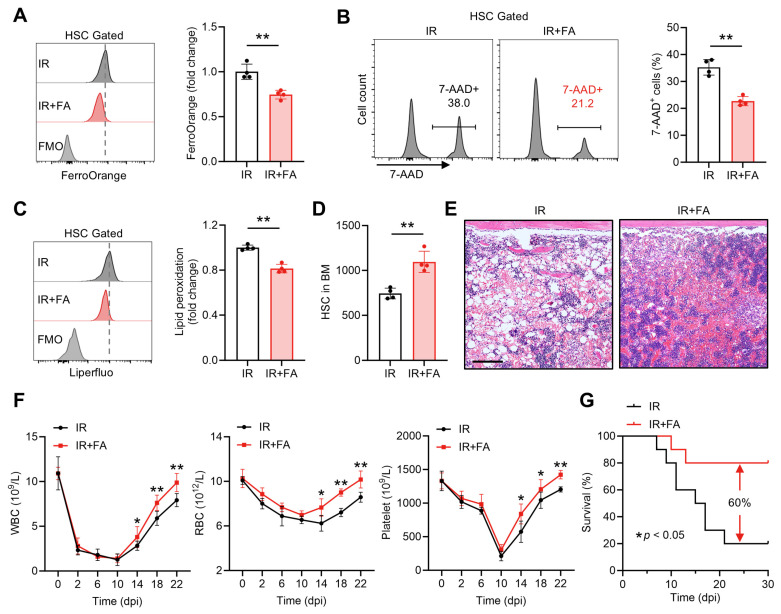
FA safeguards HSC maintenance and accelerates hematopoietic regeneration after acute myelosuppressive injury. (**A**) Flow cytometric quantification of LIP size in BM HSCs of Ctrl and FA-treated mice at 1 dpi (*n* = 4). (**B**) Flow cytometric quantification of membrane lipid peroxidation in BM HSCs of Ctrl and FA-treated mice at 1 dpi (*n* = 4). (**C**) Flow cytometric quantification of HSC death in the BM of Ctrl and FA-treated mice at 1 dpi (*n* = 4). (**D**) HSC numbers in the BM of Ctrl and FA-treated mice at 1 dpi (*n* = 4). (**E**) Representative hematoxylin and eosin staining of BM in Ctrl and FA-treated mice at 10 dpi. Scale bar, 200 μm. (**F**) WBC, RBC and platelet counts in PB of Ctrl and FA-treated mice at indicated times post IR (*n* = 6). (**G**) Survival analysis of Ctrl and FA-treated mice following lethal irradiation (*n* = 10). Data represent mean ± SD. * *p* < 0.05, ** *p* < 0.01. Two-tailed unpaired Student’s *t*-test unless stated otherwise. Kaplan–Meier survival curves and log-rank test (**G**).

We further tested whether the LIP limitation and anti-ferroptosis activity of FA are valuable for clinical translation. Acute myelosuppressive injury, in which HSC ferroptosis is implicated, is always life-threatening and a primary cause for the discontinuation of cancer therapy [5]. We then wondered if FA treatment could mitigate HSC ferroptosis and thereby rescue acute myelosuppressive injury induced by exposure to a sublethal dose (5 Gy) of ionizing radiation (IR). Consistent with previous studies [16,18], dramatic cell death (Figure S3A) and lipid peroxidation (Figure S3B) were detected in the BM HSC compartment at 1 day post IR (dpi), when HSC ferroptosis peaks, along with markedly increased LIP size (Figure S3C). Accordingly, severely impaired HSC maintenance was observed at 1 dpi (Figure S3D), accompanied by myelosuppression as manifested by dramatic reductions in WBC, RBC and platelet counts in PB (Figure S3E). However, immediate FA supplementation remarkably alleviated Fe^2+^ accumulation (Figure 7A) and ferroptosis (Figure 7B,C) of HSCs at 1 dpi. Finally, the impaired HSC maintenance (Figure 7D) and myelosuppression (Figure 7E,F) induced by IR were significantly rescued by this short-term FA supplementation regimen. Moreover, FA supplementation led to a 60% increase in survival in a murine model of lethal myelosuppressive injury induced by 7.5 Gy IR (Figure 7G). Thus, FA safeguards HSC maintenance after acute myelosuppressive injury through mitigating Fe^2+^ overload and ferroptosis of HSCs, reinforcing the promotive action of FA in HSC maintenance.

## 4. Discussion

**Figure 8:**
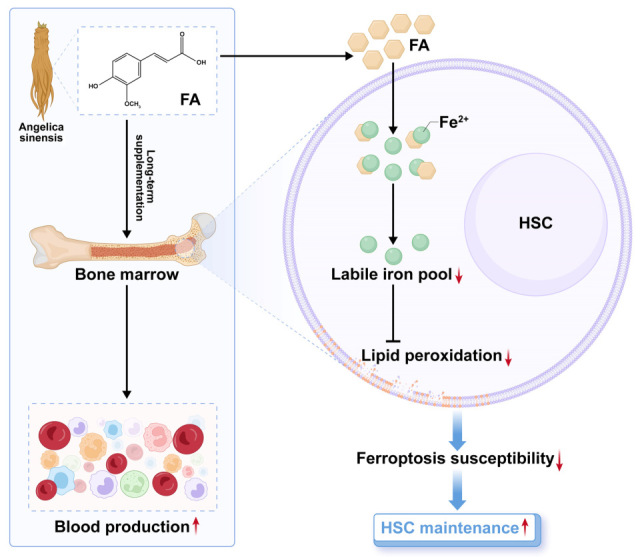
A schematic model for the pharmacological action and mechanism of FA on HSC maintenance.

In this study, we reveal that FA, a bioactive component abundantly present in Angelica sinensis, distinctively and directly promotes HSC maintenance and thereby boosts hematopoiesis in both homeostasis and acute myelosuppressive injury (Figure 8). These findings inform that the hematopoiesis-promoting activity of Angelica sinensis is partly mediated by FA through its action on HSC maintenance. Apart from Angelica sinensis, FA is also widely present in vegetables and fruits [8]. Thus, our results may partly explain why a higher intake of vegetables and fruits, a hallmark of a healthy diet pattern, benefits HSC maintenance [32]. Overall, the findings of this study substantially improve our understanding of the beneficial effect of FA-rich herbs and diet as well as suggest FA supplementation as a promising avenue to preserve functional HSCs, whose maintenance is always compromised during physiological aging [33].

Although the formulas containing FA-rich herbs are popular in traditional Chinese medicine, and are considered to offer protection against a wide array of diseases such as infection [34], cancer [35], ischemia/reperfusion injury [36], as well as kidney [37], cardiovascular [38] and neurodegenerative diseases [28], the precise mechanism of FA-endowed organ protection remains incompletely elucidated. In this study, we not only extend the therapeutic application of FA in acute myelosuppressive injury, but also demonstrate that this pharmacological effect of FA relies on its ability to diminish ferroptosis susceptibility of HSCs. Actually, numerous organ damages and degenerative pathologies such as those mentioned above are driven by ferroptosis [39]. Since that the anti-ferroptosis activity of FA is also recently reported in animal models of neural, myocardial or liver injury [29,40,41], it is tempting to speculate that ferroptosis defense acts as a common mechanism for FA-endowed organ protection. Furthermore, emerging evidence also links HSC ferroptosis to the pathogenesis of aplastic anemia and Fanconi anemia [21], implying a broader therapeutic potential of FA in BM failure syndromes.

Iron is not only fundamental for driving ferroptosis through triggering the Fenton reaction [20], but also as an essential minor mineral in the body involved in a multitude of physiological processes, including mitochondrial respiration, gene regulation and DNA synthesis or repair [42]. Thus, tight control of iron homeostasis is critical for overall health, with both iron deficiency and overload contributing to diverse pathological conditions [42,43]. Although iron is well known for its role in erythropoiesis, accumulating studies further reveal its importance in the overall hematopoiesis through regulating HSC homeostasis. For example, dietary iron deficiency enhances HSC self-renewal and expansion [44], while physiological iron loading with age drives functional decline of HSCs [24] and genetic iron overload elicits HSC maintenance impairment [45]. These lines of evidence inform that iron limitation favors the maintenance of functional HSCs, though the action mechanisms of iron in these processes are incompletely understood. We in this study show that FA is capable of limiting LIP in HSCs probably through its iron-chelating ability, thereby diminishing ferroptosis susceptibility and promoting the maintenance of functional HSCs. These data highlight a critical role of iron in dictating ferroptosis susceptibility of HSCs and a pharmacological action of FA in modulating iron homeostasis.

In addition to being an iron chelator, FA is also reported to act as a direct free radical scavenger or an indirect antioxidant by enhancing endogenous scavenging systems while inhibiting pro-oxidant enzymes [46]. Therefore, FA may be capable of promoting the elimination of free radicals that trigger lipid peroxidation. Moreover, FA is also reported to modulate the activity of multiple signaling molecules such as NRF2, which can drive resistance to ferroptosis via transcriptional activation of genes preventing lipid peroxidation and Fe^2+^ accumulation [26]. Although previous studies have documented that NRF2 activation underlies the anti-ferroptosis activity of FA in cell lines in vitro [28,30], our data indicate that the LIP limitation and anti-ferroptosis activity of FA in HSCs do not rely on NRF2 both in vivo and ex vivo. This probably reflects that the action of FA may be context-dependent or may differ between normal and malignantly transformed cells. Taken together, although we demonstrate here that FA diminishes ferroptosis susceptibility of HSCs through limiting LIP, the comprehensive mechanism underpinning the anti-ferroptosis activity of FA in different contexts and cell types still warrants further investigation.

At present, numerous ferroptosis inhibitors and antioxidants have been discovered and demonstrated for their effectiveness in preventing ferroptosis across a wide range of animal models. However, their poor efficacy and toxicity greatly limit their potential translation into clinical use [22]. Natural compounds and their derivatives continue to dominate as sources for novel therapeutic agents [47]. In this study, we demonstrate that FA as a natural product is capable of limiting LIP and diminishing ferroptosis susceptibility of HSCs, showing no obvious toxicity even with long-term in vivo supplementation. These excellent properties allow FA to be considered an attractive candidate compound for treatment of iron overload- and ferroptosis-associated diseases. Further, the molecular structure of FA is easily modified due to the presence of a variety of reactive groups. Comparing to FA, its derivatives show the characteristics of similar or even greater antioxidative activity and better stability after molecular modification [46]. All these increase the application scope for FA. For example, piperazine ferulate, a derivative of FA, has been used in China to treat glomerular diseases [48]. It is tempting to evaluate whether FA derivatives are superior in promoting HSC maintenance than FA and to search for the optimal forms of FA for different diseases in the future.

Of note, although invaluable for research, the mouse model of IR-induced acute myelosuppressive injury used in the present study has certain limitations. Crucially, species differences in radiation sensitivity [49,50] and hematopoietic dynamics [51] limit direct translation to humans. While now equipped with mechanistic insight using mouse models, the next step should be translating these findings to human subjects through well-controlled, randomized, and blinded clinical trials.

## 5. Conclusions

This study uncovers a novel pharmacological action and mechanism of FA in promoting HSC maintenance, as well as identifies FA as a LIP-limiting and anti-ferroptotic natural compound with potential as a therapeutic against acute myelosuppressive injury. The findings of this study not only aid in the understanding of the beneficial effects of FA-rich herbs and diet, but also provide a therapeutic rationale for using FA and FA-rich herbs to treat iron overload- and ferroptosis-associated pathologies.

## Data Availability

All data are available in the main text or the Supplementary Materials. Further inquiries should be directed to the corresponding author. The RNA-sequencing data are available at Gene Expression Omnibus database with accession number GSE299994 (https://www.ncbi.nlm.nih.gov/geo/query/acc.cgi?acc=GSE299994, accessed on 20 August 2025).

## Supplementary Materials

The following supporting information can be downloaded at: https://www.mdpi.com/article/10.3390/antiox14091053/s1, Figure S1: FA diminishes ferroptosis susceptibility of HSCs ex vivo; Figure S2: FA limits labile iron (Fe^2+^) pool in HSCs ex vivo independently of NRF2; Figure S3: IR induces iron accumulation, ferroptosis and maintenance impairment of HSCs; Table S1: The surface phenotypes for flow cytometry analysis of hematopoietic cell populations; Table S2: Antibodies used for flow cytometry.

## Abbreviations

The following abbreviations are used in this manuscript:

7-AAD	7-amino-actinomycin d
ANOVA	one-way analysis of variance
BM	bone marrow
CLP	common lymphoid progenitor
Ctrl	control
dpi	day post ionizing radiation
FA	ferulic acid
GMP	granulocyte/monocyte progenitor
GSEA	gene set enrichment analysis
HSC	hematopoietic stem cell
IKE	imidazole ketone erastin
IR	ionizing radiation
KEGG	Kyoto Encyclopedia of Genes and Genomes
l-FA	long-term FA supplementation
LIP	labile iron pool
MEP	Megakaryocyte–erythrocyte progenitor
MyP	myeloid progenitor
NRF2	Nuclear-factor erythroid 2-related factor 2
PB	peripheral blood
RBC	red blood cell
SD	standard deviation
s-FA	short-term FA supplementation
WBC	white blood cell
