# Supplementary material for: Ferulic Acid Promotes Hematopoietic Stem Cell Maintenance in Homeostasis and Injury Through Diminishing Ferroptosis Susceptibility
Source: Antioxidants (Basel). 2025 Aug 27;14(9):1053. doi: 10.3390/antiox14091053 (PMC12466751; doi:10.3390/antiox14091053)
Supplement: Supplementary file 1 [file antioxidants-14-01053-s001.zip › antioxidants-3811784-supplementary.pdf]

Table S1. The surface phenotypes for flow cytometry analysis of hematopoietic cell populations.

**Hematopoietic cell population**	**Surface phenotype**
Hematopoietic stem cell (HSC)	$Lineage^{-}c\text{-}Kit^{+}Sca1^{+}CD34^{-}CD135^{-}$
Myeloid progenitor (MyP)	$Lineage^{-}CD127^{-}c\text{-}Kit^{+}Sca1^{-}$
Common myeloid progenitor (CMP)	$Lineage^{-}CD127^{-}c\text{-}Kit^{+}Sca1^{-}CD16/32^{-}CD34^{+}$
Granulocyte/monocyte progenitor (GMP)	$Lineage^{-}CD127^{-}Sca1^{-}c\text{-}Kit^{+}CD16/32^{hi}$ $CD34^{+}$
Megakaryocyte-erythrocyte progenitor (MEP)	$Lineage^{-}CD127^{-}Sca1^{-}c\text{-}Kit^{+}$ $CD16/32^{-}CD34^{-}$
Common lymphoid progenitor (CLP)	$Lineage^{-}CD127^{+}Sca1^{mid}c\text{-}Kit^{+}$
Myeloid cell	$CD45^{+}Mac\text{-}1^{+}Gr\text{-}1^{+}$
B cell	$CD45^{+}B220^{+}$
T cell	$CD45^{+}CD3\varepsilon^{+}$

Table S2. Antibodies used for flow cytometry.

Antibody	Origin	Catalog number
eFluor™ 450 Mouse Hematopoietic Lineage Cocktail	eBioscience	Cat# 88-7772-72
PerCP-Cyanine5.5 Anti-Mouse Ly-6A/E (Sca-1)	eBioscience	Cat# 45-5981-82
PE-Cyanine7 anti-mouse Ly-6A/E (Sca-1)	Biolegend	Cat#25-5981-82
APC-eFluor 780 Anti-Mouse CD117 (c-Kit)	eBioscience	Cat# 47-1171-82
PE Anti-Mouse CD135 (Flt3)	eBioscience	Cat# 12-1351-83
APC Anti-Mouse CD135 (Flt3)	eBioscience	Cat# 17-1351-82
eFluor 660 Anti-Mouse CD34	eBioscience	Cat# 50-0341-82
FITC Anti-Mouse CD34	eBioscience	Cat# 11-0341-85
eFluor 450 Anti-Mouse CD45	eBioscience	Cat# 48-0451-82
FITC Anti-Mouse CD45.1	eBioscience	Cat# 12-0453-82
PE-Cyanine7 Anti-Mouse CD45.2	eBioscience	Cat# 11-0454-82
eFluor 450 Anti-Mouse CD45	eBioscience	Cat# 48-0451-82
APC-eFluor 780 Anti-human/mouse CD45R (B220)	eBioscience	Cat# 47-0452-82
PerCP-Cyanine5.5 Anti-Mouse CD3e	eBioscience	Cat# 45-0031-82
PE Anti-Mouse Ly-6G (Gr-1)	eBioscience	Cat# 12-5931-82

APC Anti-Mouse CD11b (Mac-1)	eBioscience	Cat# 17-0112-82
Anti-Ferritin Antibody [EPR3004Y]	Abcam	Cat# ab75973
NRF2 (D1Z9C) XP® Rabbit mAb (PE Conjugate)	Cell Signaling Technology	Cat#14409S
Goat anti- Rabbit IgG(H+L) Cross-Adsorbed Secondary Antibody, Alexa Fluor 488	Thermo Fisher Scientific	Cat# A-11008

Fig. S1

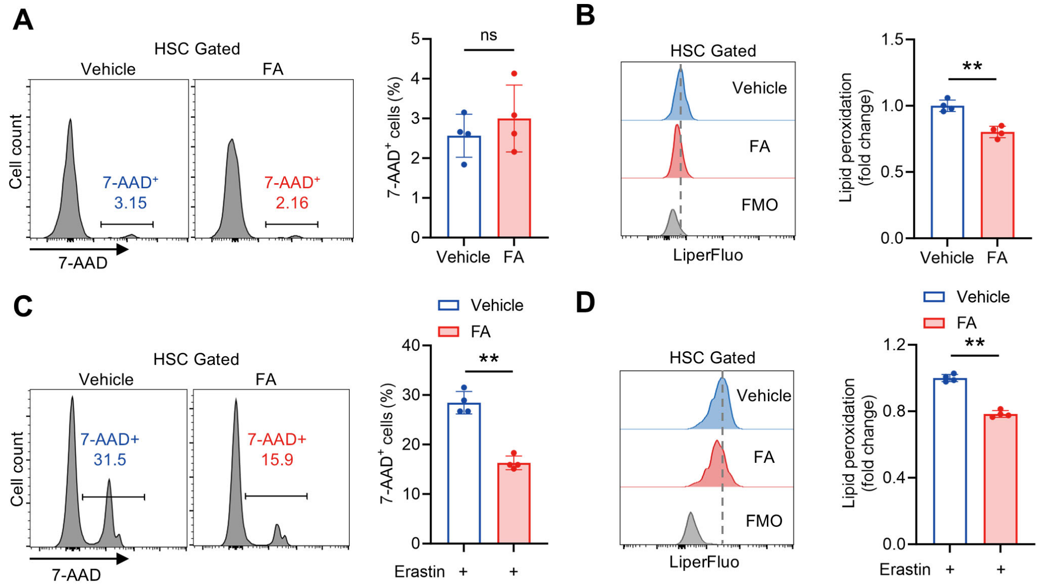

Figure S1. FA diminishes ferroptosis susceptibility of HSCs *ex vivo*. (A and B) Flow cytometric quantification of cell death (A) and membrane lipid peroxidation (B) of HSCs cultured with or without FA (5 μM; $n = 4$). (C and D) Ferroptosis susceptibility analysis of HSCs cultured with or without FA ($n = 4$). Data represent mean ± SD. $^{**}p < 0.01$. ns, no significance. Two-tailed unpaired Student's *t*-test.

Fig. S2

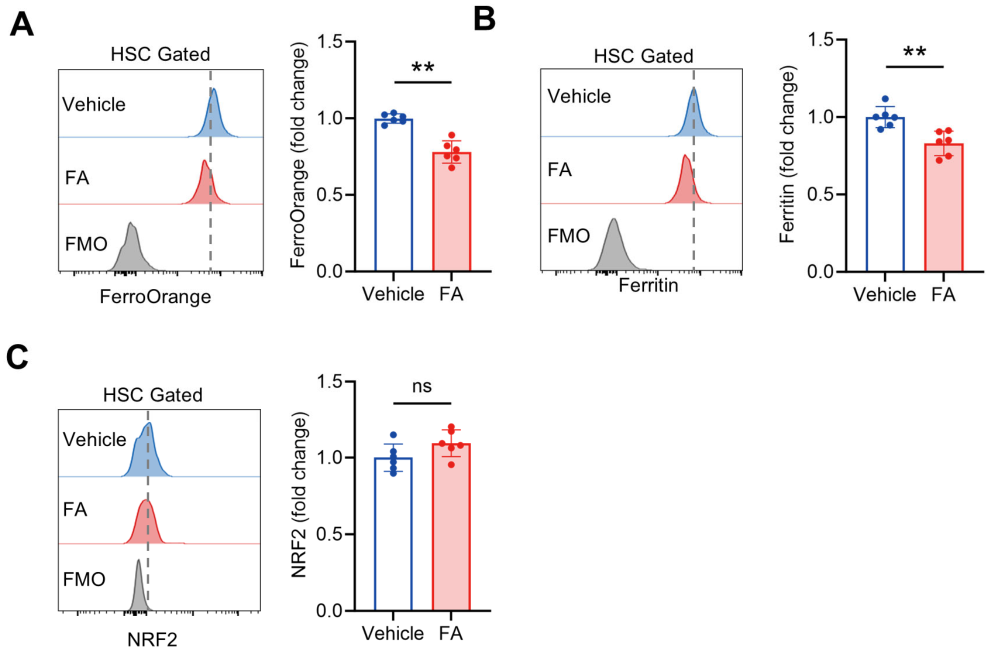

Figure S2. FA limits labile iron (Fe^{2+}) pool in HSCs *ex vivo* independently of NRF2. (A) Flow cytometric quantification of LIP size in HSCs cultured with or without FA (*n* = 6). (B) Flow cytometric quantification of ferritin expression in HSCs cultured with or without FA (*n* = 6). (C) Flow cytometric quantification of NRF2 expression in HSCs cultured with or without FA (*n* = 6). Data represent mean ± SD. ***p* < 0.01, ns, no significance. Two-tailed unpaired student's *t*-test.

Fig. S3

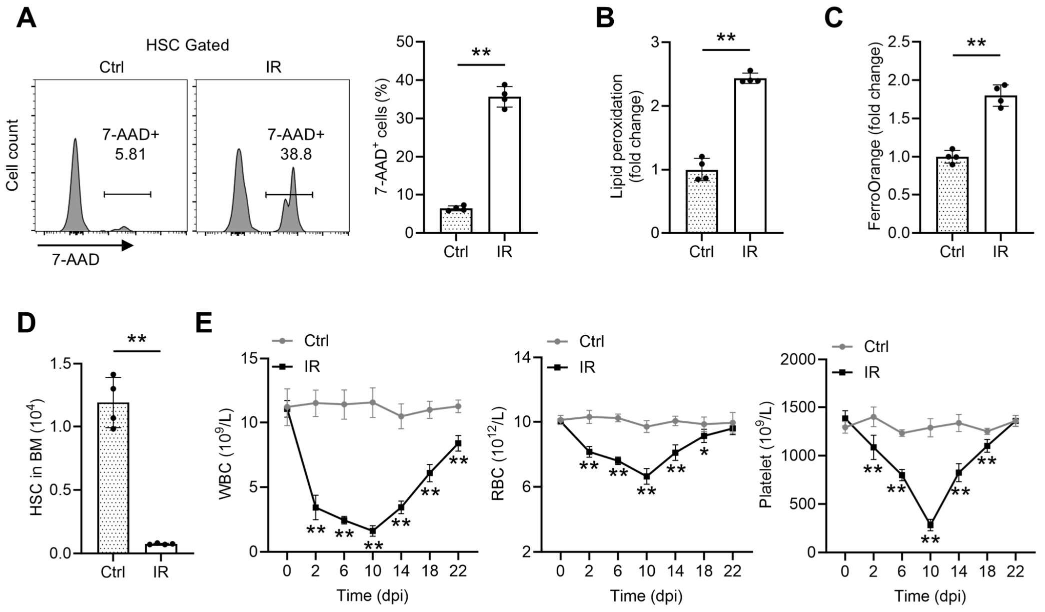

Figure S3. IR induces iron accumulation, ferroptosis and maintenance impairment of HSCs. (A and B) Flow cytometric quantification of cell death (A) and lipid peroxidation (B) in BM HSCs of mice at 1 dpi (n = 4). (C) Flow cytometric quantification of LIP size in BM HSCs of mice at 1dpi (n = 4). (D) HSC numbers in the BM of mice at 1 dpi (n = 4). (E) WBC, RBC and platelet counts in PB of mice at indicated times post IR (n = 5). Data represent mean ± SD. *p< 0.05, **p < 0.01. Two-tailed unpaired student's t-test.
